# Magnetic Nanoparticle-Integrated Microfluidic Chip Enables Reliable Isolation of Plasma Cell-Free DNA for Molecular Diagnostics

**DOI:** 10.3390/diagnostics16030460

**Published:** 2026-02-02

**Authors:** Amir Monfaredan, Sena Şen, Arash Adamnejad Ghafour, Ebru Cingöz Çapan, Muhammed Ertuğrul Çapan, Ridvan Şeçkin Özen, Şeref Buğra Tuncer, Oral Öncül

**Affiliations:** 1 Department of Molecular Medicine, School of Advanced Technologies in Medicine, Tehran University of Medical Sciences, Tehran 14177-55469, Iran; monfaredanamir@gmail.com; 2 Department of Basic Oncology, Oncology Institute, Istanbul University, 34320 Istanbul, Türkiye; sena.sen@istanbul.edu.tr (S.Ş.); pjirax@yahoo.com (A.A.G.); 3 Department of Medical Laboratory Techniques, Vocational School of Health Sciences, Avrasya University, 61080 Trabzon, Türkiye; ebru.cingoz.20@ogr.iu.edu.tr; 4 Occupational Health and Safety Program, Vocational School of Health Sciences, Avrasya University, 61080 Trabzon, Türkiye; ertugrulcapann@gmail.com; 5 Department of Medical Genetics, Faculty of Medicine, Beykent University, 34475 Istanbul, Türkiye; ridvanseckinozen@gmail.com; 6 Department of Internal Medical Sciences, Faculty of Medicine, Beykent University, 34475 Istanbul, Türkiye; 7 Department of Infectious Diseases and Clinical Microbiology, Istanbul Faculty of Medicine, Istanbul University, 34320 Istanbul, Türkiye

**Keywords:** cfDNA, DNA extraction, microfluidics, liquid biopsy, magnetic beads

## Abstract

**Background/Objectives**: Cell-free DNA (cfDNA) is a valuable biomarker for cancer diagnosis and therapy monitoring; however, its low abundance and fragmented nature present major challenges for reliable isolation, particularly from limited plasma volumes. Here, we report the development and evaluation of a novel magnetically assisted microfluidic chip with a three-inlet design for efficient cfDNA extraction from small-volume plasma samples. **Methods**: The platform enables controlled infusion of plasma, lysis buffer, and magnetic nanoparticle suspensions at defined flow rates. An external magnetic field selectively captures cfDNA-bound nanoparticles while efficiently removing background impurities. **Results**: Direct comparison with two in vitro diagnostic (IVD)-certified commercial cfDNA extraction kits showed that the microfluidic system achieved comparable cfDNA yields at standard plasma volumes and superior performance at reduced input volumes. High DNA purity and integrity were confirmed by quantitative PCR amplification of a housekeeping gene and clinically relevant targets. The complete workflow required approximately 9 min, used minimal equipment, reduced contamination risk, and enabled rapid processing with future potential for parallel multi-chip configurations. **Conclusions**: These findings establish the proposed microfluidic platform as a rapid, reproducible, and scalable alternative to conventional cfDNA extraction methods. By significantly improving recovery efficiency from small plasma volumes, the system enhances the clinical feasibility of liquid biopsy applications in cancer diagnostics and precision medicine.

## 1. Introduction

Cancer remains one of the leading causes of mortality worldwide, and early diagnosis and timely monitoring of therapeutic response are among the most important factors influencing the outcome of the disease. In this context, liquid biopsy has proven to be a revolutionary non-invasive diagnostic approach in recent years, especially in the field of oncology [1]. Liquid biopsy refers to the analysis of biomarkers such as cell-free DNA (cfDNA), extracellular vesicles, and other molecular components found in body fluids such as blood, urine, saliva, and cerebrospinal fluid, and allows clinically relevant information to be obtained without the need for invasive procedures [2].

cfDNA consists of double-stranded DNA fragments released into the bloodstream as a result of biological processes such as apoptosis, necrosis or other forms of cell death [3]. Since the quantity and structural characteristics of cfDNA are often altered in genetic diseases such as cancer, cfDNA has gained considerable attention as a valuable biomarker [4]. However, its inherently low concentration, fragmented structure, and sensitivity to environmental conditions pose major challenges for both isolation and downstream analysis [5]. Clinically relevant cfDNA is predominantly composed of short mono- and oligonucleosomal fragments (~150–600 bp), which further complicates selective isolation and increases susceptibility to genomic DNA contamination. Conventional cfDNA extraction techniques tend to be labor-intensive, time-consuming, and dependent on specialized reagents and equipment, limiting their applicability in routine clinical practice [6].

To overcome these limitations, microfluidic systems have recently gained importance as innovative alternatives for the isolation and analysis of cfDNA [7]. These technologies offer a number of advantages, including reduced sample volume requirements, faster processing times, reduced contamination risks, compatibility with automation, and portability. In addition, their ability to integrate multiple biological processes on a single platform significantly improves diagnostic and monitoring workflows [8]. At the microscale, fluid dynamics are more controllable, improving isolation accuracy and reducing operating costs [9]. Previous efforts applying nanomagnetic, size-selective platforms have demonstrated cfDNA recovery rates above 90%, with significant reduction in genomic DNA contamination, enabling improved circulating tumor DNA (ctDNA) enrichment for sequencing-based assays [10]. In contrast to batch-based silica column or magnetic bead extraction methods that rely solely on surface adsorption chemistry, microfluidic platforms enable controlled laminar flow, size-aware handling, and reduced manual intervention.

cfDNA and ctDNA analyses have been increasingly integrated with microfluidic platforms to improve the sensitivity and automation of liquid biopsy-based cancer detection from blood samples. Fully integrated microfluidic systems enable direct isolation of cfDNA from undiluted plasma using dielectrophoretic forces, followed by on-chip PCR amplification, allowing simultaneous collection, purification, and mutation detection with minimal sample volumes, including identification of clinically relevant KRAS mutations in pancreatic cancer plasma (~60 µL) [11]. Microfluidic chips incorporating magnetic bead-based or solid-phase cfDNA extraction have been further combined with on-chip quantitative PCR or droplet digital PCR to achieve automated, high-resolution mutation analysis, demonstrating applicability in ovarian, breast, and colorectal cancer patient samples [12,13,14]. Beyond PCR-based approaches, advanced signal amplification strategies, including hybridization chain reaction, catalytic hairpin assembly, and surface-enhanced Raman scattering, as well as amplification-free microfluidic biosensing systems, have enabled ultrasensitive ctDNA detection at attomolar levels, highlighting the versatility of lab-on-a-chip technologies for highly sensitive cancer diagnostics [15,16]. Collectively, these studies demonstrate that microfluidic lab-on-a-chip platforms reduce sample consumption, integrate cfDNA extraction with amplification or signal enhancement, and provide scalable solutions for sensitive and rapid ctDNA analysis in liquid biopsy applications [17,18].

In this study, we have developed a microfluidic system specifically designed for the separation of circulating nucleic acids, and we have performed both qualitative and quantitative evaluations of its performance. The system was compared to a commercially available in vitro diagnostic (IVD)-certified kit to determine the purity of cfDNA from plasma samples. In addition, the quality and integrity of cfDNA isolated by both methods were analyzed, and comparative assessments were performed. The time efficiency of each isolation method was also evaluated. A key feature of the developed platform is its specially designed channel architecture, which combines size-informed handling with magnetically assisted capture to enable selective isolation of short cfDNA fragments. This design is especially relevant for low-input samples, where conventional extraction methods often show reduced recovery efficiency and longer processing times. The observed performance at reduced plasma volumes suggests that such microfluidic approaches may be well suited for clinical settings where sample availability is limited. More broadly, microfluidic cfDNA isolation platforms offer inherent advantages, including reduced contamination risk, compatibility with automation, and modular integration with downstream analytical methods. While this study focuses on analytical performance and comparative evaluation, the modular nature of the system suggests potential for future integration with compact readout interfaces or parallelized chip designs. These characteristics highlight the relevance of microfluidic technologies as flexible upstream components within liquid biopsy workflows, supporting ongoing efforts toward streamlined and scalable cfDNA-based diagnostic applications.

## 2. Materials and Methods

### 2.1. Fabrication of the Microfluidic Chip

The mask design of the microfluidic chip was created using CorelDRAW software version 2025 (Canada). Microchannels with a length of 3 cm and a width of 100 µm were designed. The prepared pattern was transferred onto a talc-coated surface using a high-resolution printer. Subsequently, a thin layer of UV-sensitive SU-8 100 photoresist (Westborough, MA, USA) was spin-coated onto a silicon wafer by dispensing 25 mL of SU-8 at the center of the wafer and spreading it uniformly (Supplementary Methods S1). The thickness of the photosensitive layer was controlled by adjusting the spin speed using a two-step spin-coating process (500 rpm for 30 s, followed by 1500 rpm for 50 s), after which the coated wafer was cured by heating at 90 °C. The photoresist layer was then exposed to UV light in the 350–400 nm wavelength range for 4 min to induce cross-linking, followed by post-exposure baking for 2–3 min to enhance structural stability. The patterned layer was subsequently developed in an appropriate solvent for 10–15 min, rinsed with isopropyl alcohol, and dried under a nitrogen gas stream.

Polydimethylsiloxane (PDMS) and a curing agent (hardener solution) were mixed at a 10:1 ratio, poured onto the silicon mold under vacuum conditions, and cured at 80 °C for up to 30 min to obtain a transparent and elastic PDMS layer containing micron-scale structures. The microfluidic chip was fabricated using PDMS, SU-8 100 photoresist, and silica-coated Fe_3_O_4_ nanoparticles for cfDNA adsorption. The surface chemistry of PDMS was modified by plasma treatment to reduce nonspecific adsorption of proteins and other biomolecules. The microfluidic system was designed to target cfDNA fragments within the 100–600 bp size range, as confirmed by Agilent 2100 Bioanalyzer analysis (Agilent Technologies, Inc., Santa Clara, CA, USA), showing a prominent mononucleosomal peak at approximately 150–170 bp. Size-based separation within the chip was achieved through a combination of microfluidic flow dynamics and magnetically controlled capture. Similarly to conventional silica column or magnetic bead-based extraction methods, cfDNA binding was mediated by interactions with silanol groups on the surface of silica-coated Fe_3_O_4_ magnetic nanoparticles; however, the integration of microchannels enabled laminar flow, controlled mixing, and the application of a magnetic field gradient, resulting in more efficient and selective cfDNA capture (Supplementary Methods S2).

The PDMS chip mold (Sigma-Aldrich, St. Louis, MO, USA, CAS no. 63148-62-9) was fabricated using a standard soft lithography technique from a silicon wafer patterned with SU-8 100 photoresist (MicroChem Corp., Newton, MA, USA). The photoresist was spin-coated at 2300 rpm for 60 s, followed by soft baking at 65 °C for 10 min and 95 °C for 70 min. After development and isopropanol washing to remove unexposed photoresist, the wafer was post-baked to obtain a structure height of 50 µm. The microfluidic chip was designed with two chambers (G and H), each with a calculated volume of approximately 0.875 µL (dimensions: 3500 × 2500 × 100 µm), which were used for immunomagnetic particle collection and waste accumulation, respectively. The design further included two serpentine mixing channels (5000 × 500 × 100 µm), four inlets, and one outlet, with all microchannels fabricated at a uniform width of 100 µm. Following PDMS casting, the chip was plasma-bonded onto a glass slide and rendered ready for performance testing.

### 2.2. cfDNA Isolation Using Standard Kit

Ethical approval was obtained (approval date: 10 August 2023; approval number: 71304544), and the study was conducted in accordance with the Declaration of Helsinki. An a priori power analysis was conducted using the Power and Sample Size software (G*Power 3.1), with the significance level set at 0.05 and the statistical power fixed at 80%. The analysis was designed to compare two independent groups: samples processed using the newly developed method and those processed using a commercial kit. Based on an expected mean difference of 30 units between these two approaches, the minimum required sample size was calculated as 16 samples per group.

A total of 50 plasma samples used in this study were obtained from patients diagnosed with metastatic breast cancer. All participants provided written informed consent prior to sample collection, and the study protocol was approved by the relevant institutional ethics committee. Ethical approval and consent documentation were obtained in accordance with the Declaration of Helsinki. Inclusion criteria encompassed patients over 18 years of age with histopathological confirmed metastatic breast cancer who had not received systemic therapy within four weeks prior to sample collection. Exclusion criteria included the presence of other malignancies, severe comorbid conditions, or insufficient sample volume. All plasma samples were obtained via routine clinical venipuncture procedures, and no visually detectable hemolysis was observed during pre-analytical handling.

As the control group, cfDNA extraction was performed using a conventional method with the GenElute™ UltraMag Cell-Free DNA Kit (Sigma-Aldrich, USA), strictly following the manufacturer’s instructions. All samples were processed under identical volume and handling conditions and were used in comparative analyses alongside the microfluidic system.

**Figure 1:**
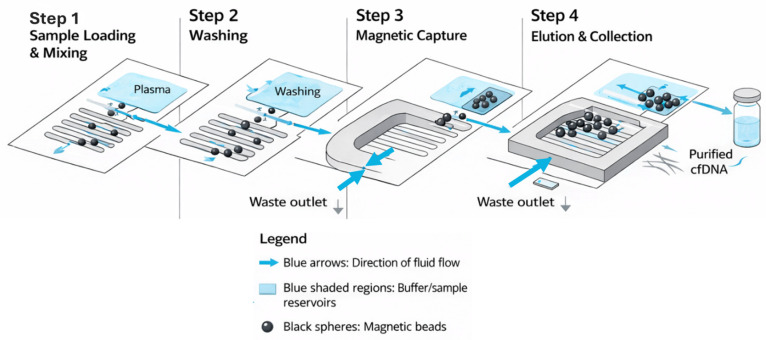
Redesigned schematic of the microfluidic chip and cfDNA isolation workflow. The figure illustrates the sequential steps of cfDNA isolation from plasma using an integrated microfluidic platform. Step 1—Sample loading and mixing: Plasma samples are introduced into serpentine microchannels, where laminar-flow–assisted mixing with functionalized magnetic beads enables efficient adsorption of cfDNA. Step 2—Washing: Washing buffers are perfused through the microchannels to remove unbound plasma components while cfDNA remains bound to the magnetic beads. Step 3—Magnetic capture: An external magnetic field is applied to immobilize cfDNA-bound magnetic beads within the capture chamber, allowing waste fluids to be flushed out. Step 4—Elution and collection: An elution buffer releases cfDNA from the beads, and purified cfDNA is collected at the outlet for downstream molecular analyses. Blue arrows indicate fluid flow direction, blue-shaded regions represent buffer or sample reservoirs, and black spheres denote magnetic beads.

The microfluidic chip was developed to enable the stepwise and controlled isolation of circulating nucleic acids. Figure 1 presents a simplified, single and integrated schematic of the microfluidic chip, in which the functional regions are numbered and indicated by directional arrows to illustrate the sequential workflow. Specifically, (i) the mixing region, where plasma interacts with surface-modified magnetic nanoparticles to facilitate cfDNA binding; (ii) the washing flow region, in which non-specifically bound biomolecules are removed under controlled laminar flow; (iii) the magnetic capture region (G chamber), where cfDNA–nanoparticle complexes are retained by an external magnetic field; and (iv) the elution region, where purified cfDNA is released and collected, are clearly delineated within the chip architecture. Panel-based subdivisions (A, B, and C) were omitted to provide a single, unified schematic representation. This streamlined microfluidic workflow enabled the efficient enrichment of short cfDNA fragments with high integrity, ensuring reliable input for subsequent quantitative and qualitative molecular analyses.

cfDNA purity was assessed by measuring A260/280 absorbance ratios using a spectrophotometer. Measurements were performed in triplicate (*n* = 3) for each extraction method. Second, qPCR performance was assessed by amplification of the *ACTB* housekeeping gene. Amplification specificity was evaluated by melt curve analysis, and amplification efficiency was determined using a log-linear standard curve generated from serial dilutions, with the coefficient of determination (R^2^) calculated. Third, fragment size distribution and contamination by high-molecular-weight DNA were analyzed using the Agilent 2100 Bioanalyzer. All cfDNA extraction experiments were performed in triplicate (*n* = 3) for each method to enable statistical comparison of extraction efficiency at a plasma input volume of 1 mL.

### 2.3. Optimization of Magnetic Nanoparticle-Based Capture

The magnetic nanoparticles used in this study were silica-coated iron oxide (Fe_3_O_4_) particles with an average diameter of 150 nm, where the silica shell provided a surface suitable for DNA adsorption in the presence of chaotropic salts. To maximize cfDNA recovery within the microfluidic chip, several parameters were optimized. The infusion rates for the plasma (10 µL/s), lysis buffer (15 µL/s), and nanoparticle suspension (5 µL/s) were adjusted to ensure rapid mixing and efficient DNA binding on contact. After initial mixing, a 5 min incubation was initiated to allow sufficient time for interaction between cfDNA and nanoparticles. The external magnetic field was then calibrated in terms of both strength and duration to ensure efficient binding of cfDNA-bound nanoparticles while removing unbound contaminants. Overall, these optimizations created a reproducible protocol that enables efficient cfDNA isolation in a streamlined and potentially automatable workflow.

### 2.4. qRT-PCR

cfDNA integrity was assessed through multi-gene amplification and fragment size profiling. Quantitative PCR was performed targeting genes of varying amplicon lengths, including ACTB, ESR1, PGR, VIM, and MKI67, to evaluate amplifiability across different fragment sizes. In addition, fragmentation profiles were analyzed using the Agilent 2100 Bioanalyzer over a size range of 35–10,000 bp. These analyses were performed to determine the suitability of the isolated cfDNA for downstream applications.

The amplification was carried out for 40 cycles with a pre-incubation at 95 °C for 15 s, followed by 10 s at 95° C, 10 s at 55° C, and 10 s at 72° C for each cycle. A melting curve was obtained by rapidly cooling down from 95° C to 65° C, followed by a 15 s incubation at 65° C and heating up to 95° C. To normalize for equal cfDNA amounts, parallel runs were conducted with actin-specific primer sets for each sample. The ΔΔCt method was used to determine target levels [19]. The following primer sets were used.

The primer sets listed in Table 1 were selected to assess both the quality and clinical relevance of the isolated cfDNA. ACTB (beta-actin) was used as a housekeeping reference gene to evaluate cfDNA integrity and enable normalization. ESR1 (estrogen receptor alpha) and PGR (progesterone receptor) are clinically relevant hormone receptor genes routinely assessed in breast cancer for prognostic evaluation and therapeutic decision-making. MKI67 (Ki-67) is a well-established proliferation marker that reflects tumor growth activity. VIM (vimentin) is a mesenchymal marker associated with epithelial-to-mesenchymal transition and metastatic potential. Successful amplification of these targets demonstrated the suitability of cfDNA isolated using the microfluidic chip for molecular oncology–relevant qRT-PCR analyses.

### 2.5. Fragment Size Distribution and Quantification of cfDNA

The cfDNA fragment size distribution and concentration were evaluated using the Agilent 2100 Bioanalyzer System with the High Sensitivity (HS) cfDNA Kit (Agilent Technologies, Santa Clara, CA, USA) according to the manufacturer’s instructions. All reagents were brought to room temperature before use. The gel–dye mixture was prepared and added to the designated chip wells. Each sample and the DNA ladder were supplemented with the provided marker solution, and 2 µL of cfDNA was added to each sample well. The chip was primed and vortexed using the Agilent IKA plate before processing on the 2100 Bioanalyzer. Data acquisition and analysis was performed using the 2100 Expert software (Part Number: G2946CA). Sizing and quantification were based on the lower (~35 bp) and upper (~10,000 bp) markers. For each sample, DNA concentration, dominant peak size, and area under the curve were plotted within predefined fragment size windows.

### 2.6. Statistical Analysis

All experimental data were analyzed with IBM SPSS Statistics (Version 30, IBM Corp., Armonk, NY, USA). Data normality was confirmed prior to statistical testing, and differences in A260/280 ratios were evaluated using appropriate parametric tests. Normality of continuous variables was tested using the Kolmogorov–Smirnov test. For normally distributed data, comparisons between groups were performed using Student’s *t*-test, while for non-normally distributed data, the Kruskal–Wallis test was used. Differences between categorical variables were analyzed using the chi-square test. Results were expressed as mean ± standard deviation (SD) or median (minimum–maximum), as appropriate. A *p*-value < 0.05 was considered statistically significant.

## 3. Results

The microfluidic chip was designed based on the evaluation of plasma viscosity–related flow characteristics. The system incorporated three distinct inlets: patient plasma was introduced through inlet A at a flow rate of 10 µL/s, lysis buffer through inlet B at 15 µL/s, and a magnetic nanoparticle-containing buffer through inlet C at 5 µL/s. Upon simultaneous activation of the three inlets, Valves 1 and 2 were initially kept closed before the magnetic field was applied beneath the chip. The plasma, lysis buffer, and nanoparticle mixture were injected into the flow channels, filling the system with a total volume of 1 mL. Following this, a 5 min incubation period was allowed without magnetic activation.

**Figure 2:**
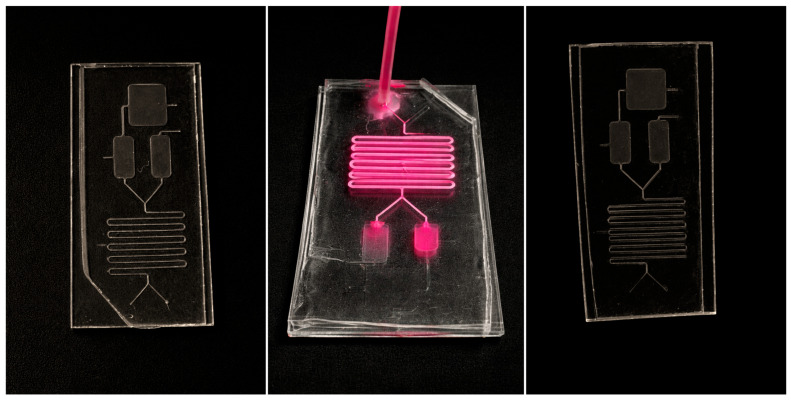
The microfluidic chip was manufactured in a clean room before drilling and testing. Activation of the external magnetic field enables selective retention of cfDNA-bound magnetic nanoparticles within the capture chamber while unbound components are removed under continuous flow.

At the end of the incubation period, Valve 1 was opened while Valve 2 remained closed, allowing the entire mixture to be directed into chamber G. During this step, valve 3 remained closed. Subsequently, the magnetic field below the chip was activated, Valves 1 and 3 were opened, and valve 4 was closed. This configuration permitted continuous fluid flow from the inlets, during which the DNA bound to the magnetic particles was retained in chamber G, while other components were flushed into chamber H (Figure 2).

Finally, valve 3 was closed while Valves 1 and 4 were opened, allowing the DNA elution buffer from inlets A, B, and C to reach the magnetic capture zone. With the magnetic field active during this stage, purified DNA was collected through outlet D. To benchmark the analytical performance of the proposed system, the microfluidic cfDNA isolation platform was directly compared with two commercially available IVD-certified extraction kits using the same plasma samples obtained from metastatic breast cancer patients. Comparative analyses demonstrated that the microfluidic platform yielded cfDNA of comparable quantity and integrity to IVD-certified methods, with consistent Ct values across tested targets. Importantly, all comparisons were performed on matched samples, ensuring that the observed differences reflected methodological performance rather than sample-related variability. In addition to analytical equivalence, the microfluidic system enabled a substantially reduced total processing time compared with standard clinical-grade extraction workflows.

**Figure 3:**
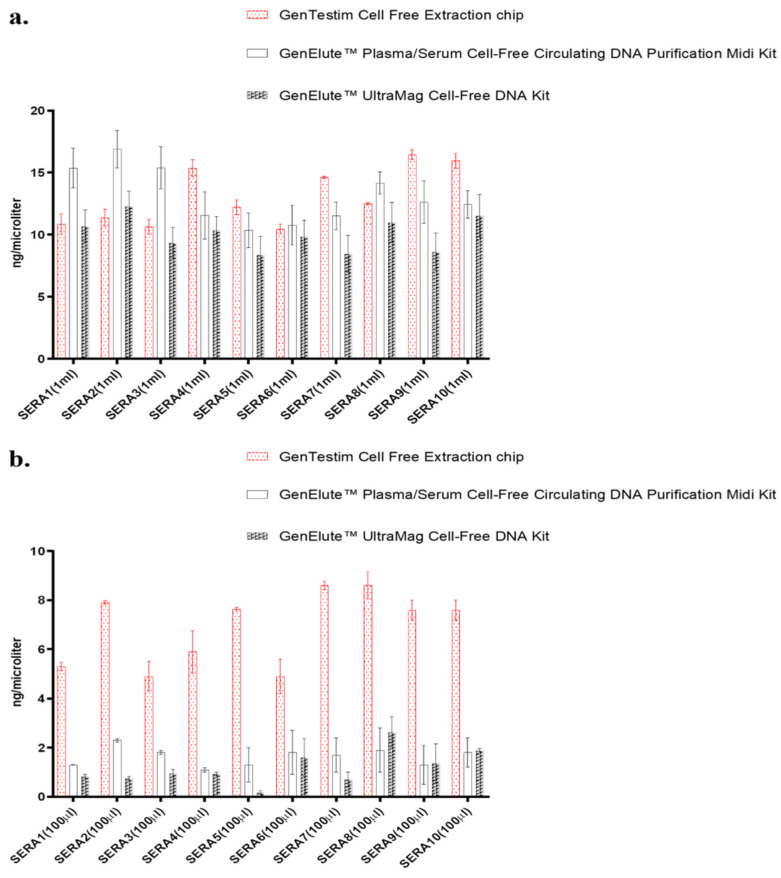
(**a**) Comparison of cfDNA yields among three methods using a 1 mL extraction volume. (**b**) Comparison of DNA yields among the three methods at reduced plasma volume (0.1 mL).

To assess the cfDNA extraction capacity of the designed chip, comparative extraction tests were performed using two different IVD-certified kits. Under a standard extraction volume of 1 mL, no statistically significant differences were observed between the three methods (Figure 3a). However, when the plasma volume was reduced to 0.1 mL, the amount of extracted DNA decreased in all three methods. Nevertheless, the microfluidic chip demonstrated superior extraction efficiency at lower volumes, supporting one of the central hypotheses of the study (Figure 3b).

### Analysis of DNA Extracted Using the Designed Chip

**Figure 4:**
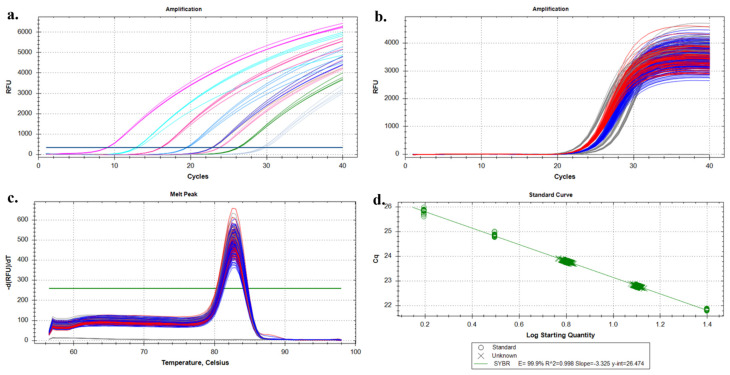
PCR performance of cfDNA extracted using the microfluidic chip targeting the ACTB gene. (**a**) Amplification curves obtained from serial dilutions of chip-extracted cfDNA, demonstrating concentration-dependent shifts in Ct values. (**b**) Amplification curves corresponding to multiple technical replicates of a single positive control (PC) concentration, illustrating assay reproducibility. (**c**) Melting curve analysis showing a single specific peak, confirming primer specificity and absence of non-specific amplification. (**d**) Standard curve generated from serial dilutions of ACTB, showing the linear relationship between Ct values and log starting quantity, used for amplification efficiency calculation. Curves are color-coded according to cfDNA concentration or replicate grouping to guide interpretation.

All cfDNA extraction experiments were performed in triplicate (*n* = 3) for each method to enable statistical comparison of extraction efficiency at a plasma input volume of 1 mL. The cfDNA extracted using the microfluidic chip was evaluated by SYBR Green-based qPCR targeting the *ACTB* gene across serial dilutions. Concentration-dependent amplification profiles were observed, with consistent Ct value across dilutions (Figure 4a), while technical replicates of a single positive control showed high reproducibility (Figure 4b). Melting curve analysis revealed a single sharp peak, indicating specific amplification (Figure 4c). The standard curve from serial dilutions showed a strong linear correlation between Ct values and log input DNA quantity, confirming reliable amplification efficiency and quantitative performance of the chip-isolated cfDNA (Figure 4d). Based on the serial dilution experiments, consistent and specific amplification was observed down to the lowest tested cfDNA input concentration. The minimum reliably detectable input corresponded to approximately 5–10 ng of DNA per reaction, as evidenced by reproducible Ct values and specific melting profiles. Accordingly, the analytical limit of detection (LOD) of the proposed microfluidic platform can be estimated to be in the low femtogram range, demonstrating its suitability for low-input liquid biopsy applications.

**Figure 5:**
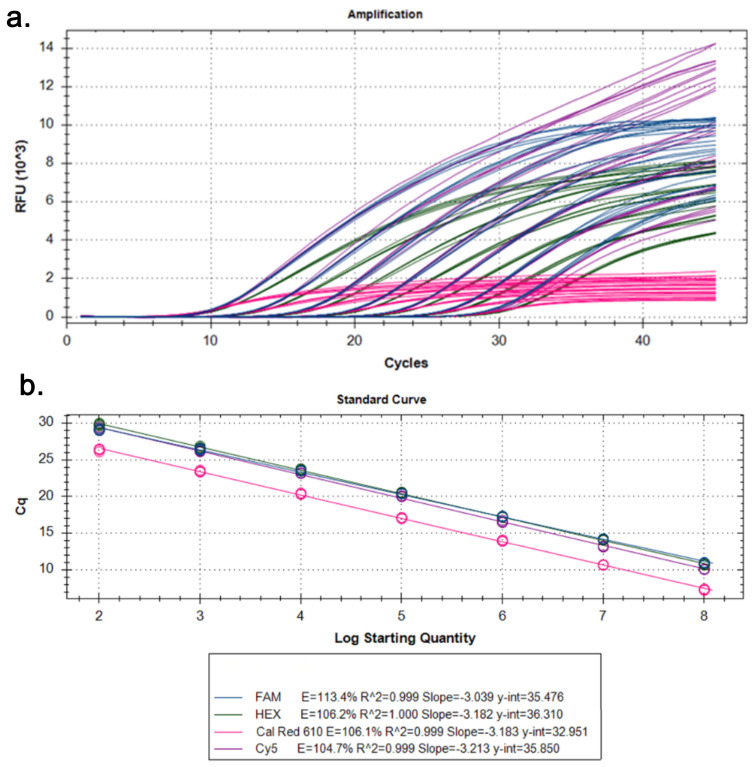
Multiplex qPCR analysis of cfDNA extracted using the microfluidic chip. (**a**) Amplification curves of ER (FAM), PR (HEX), MKI67 (Cal Red 610), and VIM (Cy5) genes obtained from chip-extracted cfDNA, illustrating target-specific amplification profiles across fluorescence channels. (**b**) Corresponding standard curves showing the linear relationship between Cq values and log starting quantity for each fluorophore, used to calculate amplification efficiency and assess quantitative performance. All targets exhibited linear amplification with high correlation coefficients, while the Cal Red 610–labeled MKI67 assay showed reduced signal intensity due to lower excitation efficiency.

To assess cfDNA integrity and multiplex compatibility, qPCR analyses were performed using fluorescent probe-based assays targeting ER, PR, MKI67, and VIM genes. Target-specific amplification curves were observed for all genes across their respective fluorescence channels, confirming the presence of amplifiable cfDNA isolated by the microfluidic chip (Figure 5a). Standard curve analysis demonstrated strong linearity between Cq values and log input DNA quantity for each target, indicating reliable quantitative performance (Figure 5b). Although the MKI67 assay labeled with Cal Red 610 showed reduced fluorescence intensity and a less pronounced amplification profile, this was attributed to lower excitation efficiency of the dye rather than compromised DNA quality.

**Figure 6:**
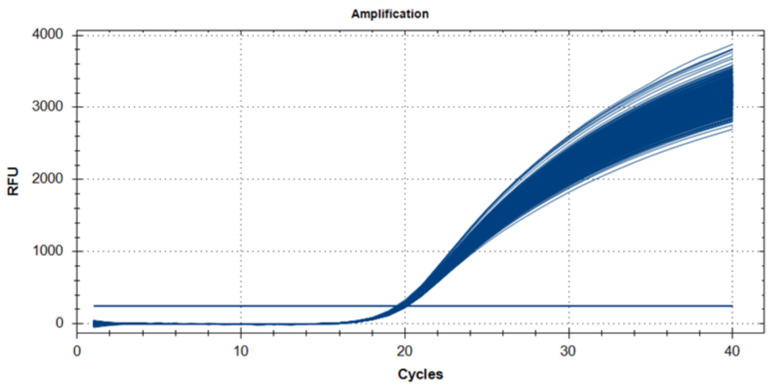
Reproducibility of DNA extraction using the microfluidic chip and qPCR validation with positive and negative controls.

For reproducibility assessment, 50 samples underwent DNA extraction in triplicate (*n* = 150 total). The resulting DNA was analyzed via qPCR using both positive and negative control templates. The data confirmed the reproducibility and reliability of the microfluidic chip system (Figure 6).

**Figure 7:**
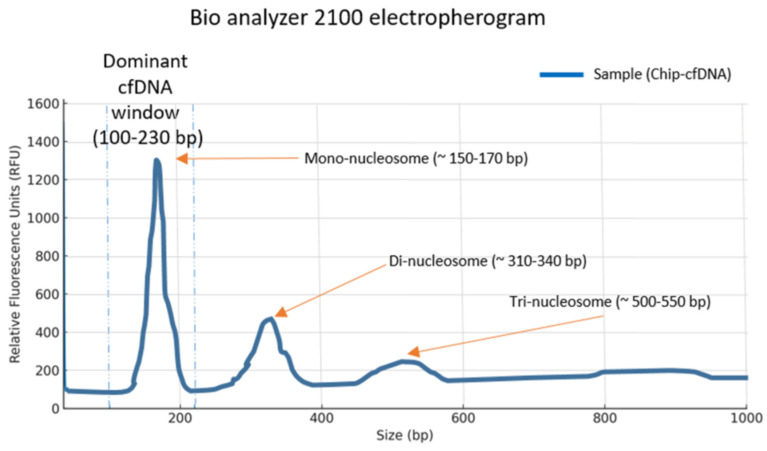
Electropherogram of cfDNA obtained using the Agilent 2100 Bioanalyzer with the High Sensitivity cfDNA kit, showing a predominant mono-nucleosomal peak (~150–170 bp) and additional di- and tri-nucleosomal fragments.

The quality and amplifiability of the cfDNA isolated using our microfluidic chip were rigorously assessed by qPCR. A standard curve constructed with serial dilutions of a control DNA template demonstrated excellent linearity (R^2^ > 0.999) and high amplification efficiency (Efficiency = 1.04618) across a dynamic range of 8 orders of magnitude (Figure 6 and Figure 7). Furthermore, the reproducibility of the extraction process was confirmed by testing a large number of replicates (*n* = 384), which showed highly consistent Ct values with a low standard deviation (Mean Ct = 19.83 ± 0.12), underscoring the robustness and reliability of our platform.

Analysis of cfDNA using the Agilent 2100 Bioanalyzer revealed a predominant mononucleosomal peak at approximately 150–170 bp, which fell within the defined cfDNA window (100–230 bp). Additional peaks corresponding to di-nucleosomal (~310–340 bp) and tri-nucleosomal (~500–550 bp) fragments were also detected, albeit with lower relative fluorescence intensities. The electropherogram showed (Figure 7) a characteristic cfDNA profile with the majority of fragments concentrated around the mononucleosomal region, and minimal background signal beyond 600 bp.

## 4. Discussion

cfDNA has emerged as an important biomarker in precision oncology, providing insights for “Point-of-Care Testing [20,21,22]. However, its low abundance and fragmentation make both its isolation and downstream analysis a technical challenge [23]. The ability to isolate cfDNA rapidly, reliably, and without contamination from limited-volume plasma samples is critical for the integration of liquid biopsy approaches into routine clinical workflows [24].

In this study, a novel microfluidic system was developed to achieve rapid, high-purity, and reliable isolation of cfDNA from blood plasma. The performance of the system was systematically evaluated and compared with two commercially available IVD-certified DNA extraction kits. As shown in Figure 1, the three-inlet design of the chip enables the simultaneous infusion of plasma, lysis buffer, and magnetic nanoparticles, creating an efficient environment for cfDNA extraction. As in the literature [25], magnetic retention zones and a gradient-based flow system improved binding efficiency and facilitated selective elution. The results show that the proposed microfluidic approach is both effective and reproducible, especially for low-volume samples where conventional methods often have reduced efficiency.

The observed A260/280 ratios (approximately 1.85–1.90) fall within the generally accepted range for high-purity DNA and are considered suitable for downstream clinical cfDNA analyses. Quantitative analysis revealed that the microfluidic system achieved significantly higher cfDNA purity (A260/280 ratio of 1.88 ± 0.03) compared to Kit A (1.72 ± 0.05) and Kit B (1.79 ± 0.04), which was statistically significant (*p* < 0.01). In addition, the total DNA yield from a 0.1 cm^3^ plasma sample was comparable between our chip (mean: 8.3 ± 0.4 ng) and Kit A (8.0 ± 0.5 ng) but was achieved in significantly less time (9 min versus 25–30 min for commercial kits). Based on serial dilution experiments, the analytical LOD was estimated to be in the low femtogram range, 5–10 ng per reaction. The consistent recovery of cfDNA from as little as 0.1 mL of plasma suggests sufficient analytical sensitivity, with recovery efficiency comparable to that of commercial kits. In addition, the use of disposable or sterilizable PDMS-based chips further reduced contamination risks. This feature is particularly relevant for pediatric oncology, rare disease cohorts, or serial monitoring where sample availability is inherently limited. The multi-inlet elution strategy ensured homogeneous buffer distribution and efficient cfDNA release, contributing to rapid recovery while preserving fragment integrity, which is advantageous for downstream molecular analyses. Furthermore, the modular architecture of the platform enables parallel processing through multi-chip configurations, supporting scalability and increased throughput for clinical and translational applications.

Downstream qPCR assays targeting the *ACTB* housekeeping gene showed robust amplification of chip-isolated cfDNA with Ct values (mean 27.6 ± 0.8) comparable to those of commercial kits (Figure 4). The qPCR results obtained in this study provide evidence for the clinical suitability of cfDNA isolated using the proposed microfluidic platform. Additionally, the qPCR performance of chip-isolated cfDNA was comparable to that obtained using IVD-certified commercial extraction kits, supporting the analytical equivalence of the microfluidic approach. These findings indicate that cfDNA isolated by the microfluidic chip meets key analytical requirements for clinically relevant liquid biopsy workflows. In addition, the integrity of the cfDNA was maintained, allowing successful amplification of clinically relevant genes such as ER, PR, and vimentin (Figure 5). The relatively weak signal for Ki67 likely reflects target-specific PCR efficiency rather than degradation due to the isolation process. These results confirm the ability of the system to maintain DNA integrity throughout the extraction process. In addition, the successful amplification of clinically relevant targets such as ER, PR, vimentin, and partially Ki67 confirmed that the extracted DNA is compatible with downstream molecular assays.

In the context of cfDNA-based liquid biopsy, the successful amplification of ACTB, ESR1, PGR, MKI67, and VIM provided important insights into both cfDNA integrity and its clinical and molecular relevance. The amplification of ACTB confirmed that the cfDNA isolated using the microfluidic platform retained sufficient integrity and amplifiability despite its fragmented nature, indicating that the extraction process did not introduce additional DNA damage. The detection of clinically relevant targets such as ESR1 and PGR demonstrated the suitability of chip-isolated cfDNA for hormone receptor–related molecular assessments, which are central to therapeutic decision-making in oncology. Furthermore, the amplification of MKI67 and VIM highlighted the ability of the platform to capture cfDNA fragments associated with tumor proliferation and epithelial-to-mesenchymal transition, respectively, suggesting that biologically informative cfDNA subsets were preserved. Collectively, these findings support the compatibility of the proposed microfluidic system with downstream qRT-PCR assays and underscore its potential applicability for clinically meaningful cfDNA-based molecular profiling.

Reproducibility, a critical factor in clinical implementation, was confirmed by triplicate extractions from 50 plasma samples. The high level of consistency observed in all qPCR runs emphasizes the potential application of the system in routine diagnostic workflows or in liquid biopsy-based precision oncology. The success of the proposed microfluidic system in low-volume and high-fragmentation environments directly addresses the current barriers to the use of cfDNA in clinical practice. Given the increasing role of cfDNA in non-invasive cancer diagnostics, therapy monitoring, and minimal residual disease detection, an efficient and scalable extraction platform is essential [26].

Our results are consistent with recent advances in microfluidics, where magnetic, electrostatic, or silica-based capture strategies have been used to improve cfDNA recovery [27,28,29,30,31,32,33]. Methods such as electrophoresis and covalent binding have shown high efficiency but often require complex chip fabrication and are prone to electrochemical degradation [34]. In contrast, existing platforms are often limited by complexity and low throughput, whereas our system overcomes these barriers through cost-effective PDMS fabrication and a parallelizable design, facilitating easier adoption into clinical laboratories. In contrast, the design of our chip enables reusable, low-cost production with consistent performance and no detectable cross-contamination [35]. Whereas the nano-magnetic size-selective platform previously reported achieves >90% cfDNA recovery and notable ctDNA enrichment [10], the present microfluidic design offers additional advantages, including reduced sample volume requirements, minimized contamination risk, and compatibility with high-throughput processing. As shown in Figure 4, our prototype can be adapted to fluorescence-based quantification modules, allowing for future automation. Importantly, the comparative study conducted here serves as a benchmark for further standardization. While commercial kits remain the clinical standard, their limitations in Point-of-Care Testing applications (due to processing time, reagent cost, and input volume) are clearly overcome by our microfluidic platform [18]. Unlike batch-based extraction methods, the proposed microfluidic design supports the potential for parallelization through future multi-channel or multi-chip architectures and may be adaptable for integration into automated diagnostic workflows; however, the current prototype processes a single sample sequentially. This feature not only reduces the time required, but also positions the system as a scalable solution for routine clinical use.

Beyond analytical performance, several important translational implications can be identified for the proposed system [26]. Due to its simplicity and minimal laboratory infrastructure requirements, the platform is considered suitable for adaptation to point-of-care settings [22]. Although regulatory validation and large-scale clinical studies are still pending, the performance characteristics demonstrated here indicate the potential of this microfluidic system to combine experimental innovation with clinical translation in precision oncology.

Notably, the comparison with IVD-certified commercial cfDNA extraction kits was not exploratory but constituted an integral part of the present study, demonstrating that the proposed microfluidic platform achieves comparable analytical performance while significantly reducing total processing time. To further contextualize the performance of the proposed microfluidic platform, a comparative analysis with previously reported microfluidic cfDNA/ctDNA isolation technologies was performed (Table 2). Existing platforms employ a wide range of separation principles, including dielectrophoresis, solid-phase surface adsorption, magnetic bead-based capture, and advanced signal amplification strategies, often integrated with on-chip detection modules. While several of these systems achieve ultra-high analytical sensitivity or direct mutation detection, they typically require longer processing times, increased system complexity, or specialized instrumentation. In contrast, the platform presented in this study was specifically optimized for rapid and robust cfDNA isolation, demonstrating efficiency comparable to IVD-certified kits and superior performance at low plasma input volumes (0.1 mL), while maintaining a substantially reduced processing time (~9 min). As summarized in Table 2, the modular and scalable design of the present system distinguishes it from more complex integrated approaches, positioning it as a flexible upstream module that can be readily combined with downstream qPCR, ddPCR, or sequencing-based assays for precision oncology applications.

### 4.1. Clinical Translation and Regulatory Considerations

Although not implemented in the current study, integrating a smartphone-based interface is a potential future extension of the proposed microfluidic platform. In principle, smartphone-assisted image processing could be used for fluorescence-based signal readout, timing control, and digital data acquisition, enabling portable and point-of-care-compatible operation without altering the core microfluidic workflow. Analytical validation studies should formally define key performance parameters, accuracy, precision, specificity, and linearity. Clinical validation should then be performed through multicenter studies involving diverse cancer types and appropriate healthy control cohorts to assess clinical sensitivity and specificity across different use cases. After successful analytical and clinical validation, regulatory approval pathways such as CE-IVD marking (Europe) or FDA 510(k) clearance (United States) will be necessary. In parallel, manufacturing processes must be standardized in compliance with ISO 13485 and Good Manufacturing Practice (GMP) requirements to ensure reproducibility, quality control, and regulatory compliance for routine clinical use.

### 4.2. Limitations

While microfluidic technologies, in general, face challenges related to yield, cost, and standardization for clinical cfDNA isolation, our platform directly addresses several of these limitations. The three-inlet design with magnetically assisted capture mitigates non-specific binding and enhances purity, effectively handling the challenges of low concentration and fragmented cfDNA. Furthermore, the use of soft lithography with PDMS offers a cost-effective and reusable fabrication route, alleviating concerns about high production costs. Although broader standardization remains a field-wide challenge, the reproducibility and reliability demonstrated here across 50 clinical samples are a significant step towards protocol harmonization. Several limitations of the proposed microfluidic system should be acknowledged. Manual valve control currently limits automation and scalability. In addition, the PDMS fabrication process requires further optimization for large-scale production. Maintaining a homogeneous magnetic field distribution across multiple microchannels remains a technical challenge and may affect capture consistency. The current prototype is limited to processing a single sample per run, which restricts throughput in its present configuration. In addition, while PDMS-based fabrication enables rapid prototyping and low initial costs, further optimization and standardization will be required to support large-scale manufacturing and routine clinical deployment. Although the per-chip material cost is relatively low, additional expenses associated with magnetic nanoparticles and reagents should be considered when evaluating overall cost efficiency. Looking ahead, we anticipate that further innovations—such as optimizing the surface chemistry of our magnetic nanoparticles, parallelizing the chip design for high-throughput screening, and potentially integrating AI for real-time process control—will be built upon this foundational design. These future directions, based on our current prototype, hold the promise to not only overcome existing barriers but also to firmly establish our specific microfluidic approach as a robust and gold-standard method for cfDNA isolation in precision medicine.

### 4.3. Future Directions

While qPCR validation confirmed the high quality and amplifiability of the isolated cfDNA, future studies will focus on validating the clinical utility of our platform through NGS. In addition, the integration of unique molecular identifier-based error correction strategies with digital PCR and NGS could further enhance mutation detection sensitivity and allow robust ctDNA profiling for applications such as minimal residual disease monitoring. This approach will enable the detection of clinically relevant mutations at low levels as found in ctDNA, demonstrating the ability of our system to increase the sensitivity of variant detection. The integration of our microfluidic chip with sequence-based assays will enable a more comprehensive evaluation of its translational potential in precision oncology, including applications for minimal residual disease detection and therapy monitoring.

Future development of the platform will focus on increasing throughput through parallelization strategies, including the integration of multiple parallel microchannels within a single chip or the implementation of multi-chip array configurations. Moreover, large-scale fabrication is expected to further reduce the per-chip cost, potentially making the system more cost-effective than commercial cfDNA extraction kits, which typically cost USD 20–50 per sample. Such approaches would enable simultaneous processing of multiple samples without substantially increasing workflow complexity. Moreover, large-scale fabrication is expected to further reduce the per-chip cost, potentially making the system more cost-effective than commercial cfDNA extraction kits, which typically cost USD 20–50 per sample. These advancements may facilitate the translation of the proposed microfluidic system into high-throughput clinical and research settings, particularly in resource-limited environments.

For clinical translation, a stepwise validation and regulatory pathway is required.

## 5. Conclusions

In summary, we evaluated the purity, integrity, quality, and processing time of cfDNA isolation by comparing a newly developed microfluidic system with a commercially available IVD-certified standard kit. The results demonstrated that the microfluidic platform enables cfDNA isolation with higher purity and in a shorter time. Furthermore, the system’s ability to operate with low sample volumes and its compatibility with integrated analytical workflows highlight its potential to enhance the accessibility and efficiency of cfDNA-based liquid biopsy applications in clinical settings. The custom-designed channel system, which combines size-based separation with a smart conductivity principle governed by a magnetic field, offers an innovative and sustainable alternative for cfDNA isolation. Future studies involving different patient cohorts and integration with diverse molecular assays may further expand the utility of this approach in precision oncology.

## Figures and Tables

**Table 1:** Primer sequences used in this study (5′→3′).

Gene Symbol	Primer Name	Sequence (5′→3′)
*VIM*	Forward	AACTTAGGGGCGCTCTTGTC
	Reverse	ATTCAAGTCTCAGCGGGCTC
*ACTB*	Forward	CCTCGCCTTTGCCGATCC
	Reverse	CGCGGCGATATCATCATCC
*ESR1*	Forward	CTCTAACCTCGGGCTGTGC
	Reverse	AGATGCTTTGGTGTGGAGGG
*MKI67*	Forward	CTGACCCTGATGAGAGTGAGGG
	Reverse	AGTTCAGGTCTTAGGTGCCC
*PGR*	Forward	AAATCTACAACCCGAGGCGG
	Reverse	CTCTCGGTACAGCCCATTCC

**Table 2:** Comparative overview of microfluidic platforms for cfDNA isolation and analysis.

Platform/Method	Separation Principle	Sample Volume (mL)	Processing Time (min)	Efficiency/ Recovery	Limit of Detection (LOD)	On-Chip Detection	Sample Type (Study)	Scalability/Parallelization
This Study	Magnetic nanoparticles, tri-inlet microfluidic flow	0.1–1.0	~9	Comparable to IVD kits at 1 mL; ~12–18% higher yield at 0.1 mL	5–10 ng per reaction	No (off-chip qPCR)	Human plasma (metastatic breast cancer)	High potential (modular, parallelizable design)
DEP-based integrated system [11]	Dielectrophoresis + on-chip PCR	~0.06	~30–40	High (integrated capture + amplification)	KRAS mutations detected at low allele fractions	Yes (on-chip PCR)	Human plasma (pancreatic cancer)	Limited (complex integration)
Magnetic bead microfluidic chip + qPCR [14]	Magnetic bead capture + on-chip qPCR	~0.2–1.0	~30	High, automated extraction and detection	Mutation-level (BRCA1)	Yes (on-chip qPCR)	Human plasma (ovarian cancer)	Moderate
Solid-phase μSPE microfluidic chip [13]	Surface-functionalized solid phase binding	~0.2–1.0	~20–30	High across cfDNA fragment sizes	Mutation-level (KRAS)	Partial (downstream detection)	Human plasma	Moderate
Magnetic extraction + ddPCR [12]	Dynamic magnetic capture + ddPCR	~0.5–1.0	~40–60	High-resolution quantitative recovery	Very low (ddPCR-based)	Yes (ddPCR)	Human serum (breast, colon cancer)	Limited
SERS-based microfluidic biosensor [16]	CHA/HCR cascade + SERS signal amplification	<0.1	~15–30	Ultra-high analytical sensitivity	Attomolar range	Yes (SERS)	Human plasma (gastric cancer)	High (chip array–based)
Nano-magnetic size-selective cfDNA capture system [10]	Nano-magnetic, size-selective capture	1–2	~60	>90% cfDNA recovery	Not reported	No	Human plasma	Limited
DEP-based Chip [36]	Dielectrophoresis	0.5–1	30–45	High purity	Not reported	No	Spiked serum	Complex, low
Silica Membrane Microchip [37]	Surface adsorption-based binding	1–3	~30	Good recovery	Not reported	No	Human plasma	Moderate

## Data Availability

The data are not publicly available due to ethical restrictions and patient privacy concerns. The dataset used during the current study is available from the corresponding author upon reasonable request.

## Supplementary Materials

The following supporting information can be downloaded at: https://www.mdpi.com/article/10.3390/diagnostics16030460/s1, Supplementary Methods S1. Microfluidic Mask Design and Photolithography Parameters. Supplementary Methods S2. Detailed Microfabrication and Chip Design Rationale.
