# Supplementary material for: Magnetic Nanoparticle-Integrated Microfluidic Chip Enables Reliable Isolation of Plasma Cell-Free DNA for Molecular Diagnostics
Source: Diagnostics (Basel). 2026 Feb 2;16(3):460. doi: 10.3390/diagnostics16030460 (PMC12896869; doi:10.3390/diagnostics16030460)
Supplement: Supplementary file 1 [file diagnostics-16-00460-s001.zip › diagnostics-4003289-supplementary.pdf]

Supplementary Methods S1. Microfluidic Mask Design and Photolithography Parameters

1. The microfluidic mask was designed using CorelDRAW rather than specialized microfabrication software to enable rapid and cost-effective prototyping with broad accessibility. The vector-based drawing environment allowed high-resolution pattern definition and reduced design iteration time compared with conventional lithography software. A channel width of 100 μm was selected to ensure stable laminar flow conditions, minimize particle clogging, and optimize the surface-to-volume ratio for efficient cfDNA capture. At this scale, low Reynolds number flow supported controlled mixing and size-selective, magnetically assisted separation.

2. Prior to pattern transfer, the substrate surface was coated with talc powder to prevent adhesion of the UV mask and to facilitate clean mask removal, thereby improving pattern fidelity and fabrication reproducibility. The accuracy of the transferred microchannel patterns was strongly influenced by printer resolution; high-resolution printing (≥2400 dpi) ensured sharp feature edges and dimensional accuracy, whereas lower resolutions resulted in increased variability in channel width.

3. SU-8 100 photoresist was selected due to its ability to form thick layers (~50 μm) with high aspect ratios, as well as its chemical and mechanical stability, which are essential for robust master mold fabrication. In addition, its low optical scattering properties enabled efficient deep-UV exposure and uniform crosslinking. The final SU-8 layer thickness was measured and verified using a profilometer or scanning electron microscopy (SEM) to ensure consistency across fabricated masters.

4. A two-step spin-coating process was employed to achieve uniform SU-8 layer formation and precise thickness control. An initial spreading step at 500 rpm facilitated homogeneous distribution of the photoresist across the substrate, followed by a higher-speed step at 1500 rpm to achieve the target thickness of approximately 50 μm with a smooth surface profile. The coated structures were subjected to three successive soft-bake steps at 90 °C to promote solvent evaporation, reduce internal stress, and enhance resist stability, thereby minimizing crack formation. UV exposure parameters were experimentally optimized within the 350–400 nm wavelength range, corresponding to the photochemical activation band of SU-8. An exposure duration of 4 minutes was selected to ensure sufficient crosslinking while avoiding under- or over-exposure that could compromise pattern fidelity.

Supplementary Methods S2. Detailed Microfabrication and Chip Design Rationale

1. UV Exposure, Post-Exposure Bake, and Development Conditions

Insufficient UV exposure of SU-8 photoresist results in incomplete crosslinking, leading to structural degradation or feature loss during the development process. Conversely, excessive exposure promotes lateral over-crosslinking, which reduces pattern resolution and causes distortion of microchannel dimensions. To enhance polymer network formation, a post-exposure bake (PEB) step was applied at 90 °C for 2–3 minutes, facilitating the completion of radical-mediated crosslinking reactions and improving mechanical stability. Development was performed using propylene glycol methyl ether acetate (PGMEA), with a controlled duration of 10–15 minutes. Shorter development times may leave residual unexposed resist, while prolonged exposure to the developer can cause excessive feature erosion and reduced resolution.

2. PDMS Preparation and Curing Parameters

Polydimethylsiloxane (PDMS) base and curing agent were mixed at a standard 10:1 (w/w) ratio to achieve an optimal balance between elasticity and mechanical strength suitable for microfluidic applications. The mixture was degassed under vacuum to eliminate entrapped air bubbles, thereby improving optical transparency and ensuring uniform channel formation. Curing was carried out at 80 °C, which accelerates polymerization while preserving the mechanical integrity and transparency of the PDMS chip. SU-8 100 was selected to achieve a feature height of 50 μm due to its high viscosity and suitability for thick-film lithography. Spin-coating speed was the primary parameter controlling film thickness, with lower speeds yielding thicker layers.

3. Soft Bake, Hard Bake, and Thickness Verification

A two-step soft bake protocol was applied to control solvent evaporation and minimize internal stress within the SU-8 layer. An initial bake at 65 °C enabled gradual solvent removal, reducing the risk of crack formation, followed by a second bake at 95 °C to remove residual solvent and stabilize the resist. After development, a hard bake at 150 °C was performed to further strengthen the crosslinked polymer network, enhance surface hardness, and improve mold durability for repeated PDMS casting. The final feature height of 50 μm was verified using profilometry or scanning electron microscopy (SEM).

4. Development Verification and Channel Design Strategy

Following development, isopropanol rinsing was used as a qualitative indicator to confirm the complete removal of unexposed SU-8, with the absence of white residues indicating successful development. Verification was further supported by microscopic inspection and profilometric measurements. The microfluidic design incorporated two immunomagnetic collection chambers to improve separation efficiency and prevent cross-contamination. One chamber was designated for capturing DNA–nanoparticle complexes, while the second served as a waste and washing reservoir. Serpentine channels were employed instead of straight channels to enhance mixing efficiency under laminar flow conditions by inducing secondary flow patterns.

5. Serpentine Channel Geometry and Chip Architecture

The serpentine channels were designed with dimensions of 5000 × 500 × 100 μm (length × width × height), providing sufficient residence time for efficient mixing while maintaining manageable

pressure drops and minimizing clogging risk. The chip architecture included four inlet ports and a single outlet to enable independent and controlled introduction of plasma samples, lysis buffer, and nanoparticle suspensions, as well as efficient collection of the eluate. The immunomagnetic chamber dimensions (3500 × 2500 × 100 μm) were optimized to ensure adequate particle retention time within the magnetic field region, thereby enhancing capture efficiency.

6. Plasma Bonding Stability and System Scalability

PDMS layers were irreversibly bonded using oxygen plasma activation (O_2 plasma, 50 W, 30 s). The resulting plasma-bonded interfaces remained stable under continuous flow and moderate pressure conditions for up to two weeks. To ensure proper channel alignment and leak-free sealing, plasma treatment was combined with controlled mechanical compression during bonding. The modular chip architecture supports scalability through parallel chip arrays and facilitates integration with automated pumping and valving systems, enabling compatibility with automated diagnostic platforms and computer- or smartphone-controlled operation.

Flow Control, Magnetic Separation, and Elution Strategy

7. Flow Rate Optimization and Valve Control Strategy

The flow rates for plasma (10 μL/s), lysis buffer (15 μL/s), and magnetic nanoparticle suspension (5 μL/s) were empirically optimized to balance efficient lysis, controlled mixing, and stable nanoparticle–DNA binding while preventing channel clogging. A slower plasma flow minimized shear-induced aggregation and obstruction, whereas a higher lysis buffer flow facilitated rapid cell-free nucleoprotein disruption and protein removal. The nanoparticle suspension was introduced at a lower flow rate to promote controlled interaction and binding kinetics. The resulting inlet flow ratio of 1:1.5:0.5 enabled effective mixing while maintaining optimal pH and ionic strength conditions for cfDNA adsorption onto magnetic nanoparticles. Fluid routing within the chip was regulated using pneumatic membrane valves, which could be operated either manually or through automated pressure control, allowing precise temporal regulation of each process step.

8. Filling, Incubation, and Magnetic Field Timing

During the initial filling phase, valves 1 and 2 were kept closed to ensure bubble-free priming of the microchannels and uniform reagent distribution. A 5-minute incubation period was selected based on experimentally determined binding kinetics, where shorter durations resulted in incomplete cfDNA–nanoparticle association and longer times did not yield significant additional binding. Importantly, no magnetic field was applied during incubation to allow free diffusion of nanoparticles, promoting homogeneous interaction with cfDNA throughout the reaction volume. Early magnetic confinement was avoided, as it led to premature aggregation and reduced binding efficiency.

9. Magnetic Capture and Washing Configuration

Selective retention of cfDNA-bound magnetic nanoparticles was achieved by activating a neodymium magnet (~0.5 T) positioned beneath the designated capture chamber. The applied magnetic field generated a sufficient gradient to induce magnetophoresis of Fe_3O_4-based nanoparticles while allowing unbound components to be flushed away. To minimize particle loss during washing, the chamber geometry was optimized to enhance magnetic field exposure, and washing steps were performed at low flow rates (5–10 μL/s). During this phase, valves 1 and 3 were opened while valve 4

remained closed, directing flow through the capture chamber and enabling continuous washing without disrupting magnetic retention of the nanoparticle–DNA complexes.

10. Cross-Contamination Prevention and Elution Strategy

Cross-contamination between the capture (G) and waste (H) chambers was minimized through a combination of physical separation via narrow interconnecting channels and sequential washing steps to remove residual contaminants. During cfDNA recovery, elution buffer was introduced simultaneously through all three inlets (A, B, and C), ensuring homogeneous buffer distribution and stable pH conditions throughout the chamber. The elution buffer composition was optimized using a low-salt formulation (e.g., TE buffer, pH 8.0) to weaken electrostatic interactions between cfDNA and the nanoparticle surface. Mild heating (60–65 °C) was applied to accelerate DNA desorption and enhance recovery efficiency without compromising fragment integrity.
